# Biophysical induction of cell release for minimally manipulative cell enrichment strategies

**DOI:** 10.1371/journal.pone.0180568

**Published:** 2017-06-30

**Authors:** Pascal Joly, Thomas Schaus, Andrea Sass, Anke Dienelt, Alexander S. Cheung, Georg N. Duda, David J. Mooney

**Affiliations:** 1 Wyss Institute for Biologically Inspired Engineering at Harvard University, Boston, United States of America; 2 Julius Wolff Institute and Center for Musculoskeletal Surgery, Charité - Universitätsmedizin Berlin, Berlin, Germany; 3 Berlin-Brandenburg Center for Regenerative Therapies, Charité-University Medicine, Berlin, Germany; 4 School of Engineering and Applied Sciences, Harvard University, Cambridge, United States of America; Centro Cardiologico Monzino, ITALY

## Abstract

The use of autologous cells harvested and subsequently transplanted in an intraoperative environment constitutes a new approach to promote regeneration. Usually cells are isolated by selection methods such as fluorescence- or magnetic- activated cell sorting with residual binding of the antibodies or beads. Thus, cell-based therapies would benefit from the development of new devices for cell isolation that minimally manipulate the target cell population. In the clinic, 5 to 10 percent of fractures do not heal properly and CD31+ cells have been identified as promising candidates to support bone regeneration. The aim of this project was to develop and prototype a simple system to facilitate the enrichment of CD31+ cells from whole blood. After validating the specificity of a commercially available aptamer for CD31, we combined this aptamer with traditional magnetic bead strategies, which led to enrichment of CD31+ cells with a purity of 91±10%. Subsequently, the aptamer was attached to agarose beads (Ø = 100–165 um) that were incorporated into a column-based system to enable capture and subsequent release of the CD31+ enriched cells. Different parameters were investigated to allow a biophysical-based cell release from beads, and a simple mixing was found sufficient to release initially bound cells from the optimized column without the need for any chemicals that promote disassociation. The system led to a significant enrichment of CD31+ cells (initial population: 63±9%, released: 87±3%) with excellent cell viability (released: 97±1%). The composition of the released CD31+ fraction indicated an enrichment of the monocyte population. The angiogenic and osteogenic potential of the released cell population were confirmed in vitro. These results and the simplicity of this system highlight the potential of such approach to enable cell enrichment strategies in intraoperative settings.

## Introduction

**Fig 1:**
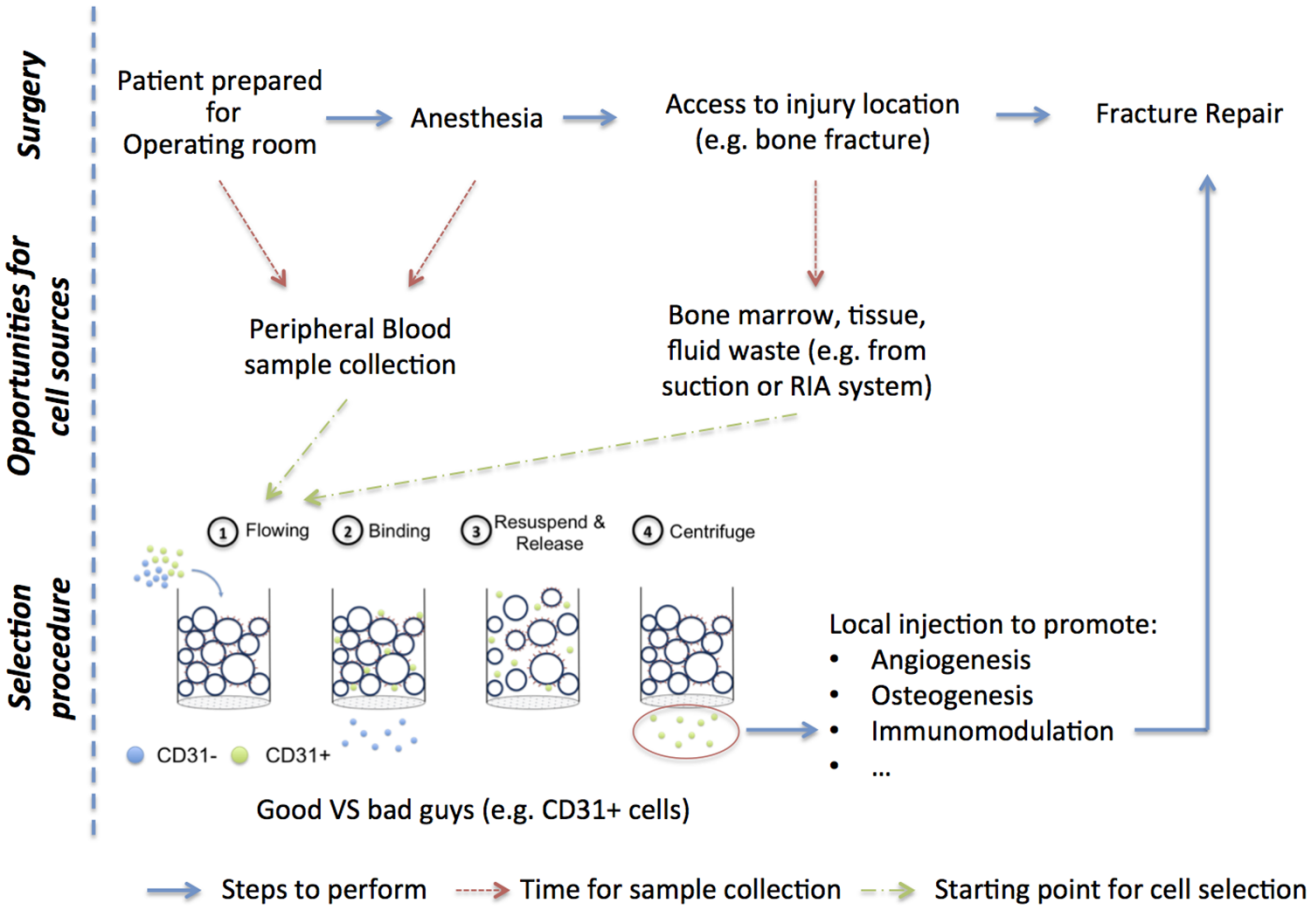
General concept of intraoperative cell therapies and its application for bone fracture. While the patient is being prepared for surgery or just after anesthesia, a blood sample is collected. Other sources such as bone marrow, hematoma or tissue waste have also the possibility to be collected intraoperatively. In parallel to the surgeon accessing the injury location (e.g. bone fracture), a specific cell population from the sample is the target of enrichment or depletion. A general outline is illustrated using CD31+ cells as an example. The desired fraction is then ready to be injected locally to promote a regenerative process. The duration of the enrichment procedure should coincide to the time required to access the location of the injury.

In order for cell therapies to be translated from the bench to the clinic, they must follow good manufacturing practice guidelines and be approved by regulatory agencies. In the case of exogenous cell therapies, significant regulatory constraints on cell isolation and in vitro expansion procedures to ensure the quality and safety of the resultant product lead to high costs [1,2]. An alternative approach to obtain a sufficient number of cells is cytokine-based cell mobilization, such as the use of granulocyte colony-stimulating factor for the mobilization of hematopoietic stem cells [3]. However, not only does this approach necessitate several visits to the hospital for the initial injections or to collect the cells, but is also associated with a wide variety of side-effects ranging from flulike symptoms to more severe conditions [4]. In contrast, intraoperative cell therapies, in which cells are harvested from the patient prior or during the initial operation and then re-administered during the same surgical session, represent a new class of exciting approaches that hold promise to overcome the high costs and many of the potential drawbacks associated with ex vivo cell expansion, cytokine-based cell mobilization, and save time for both patients and clinicians (Fig 1). Bone healing may be an ideal candidate to illustrate such a concept: in the US, approximately 7.9 million bone fractures are reported each year with 5 to 10% resulting in an impaired bone-healing situation [5,6]. Predicting patients at risk and initially providing them with additional treatment may significantly reduce the number of non-union cases and decrease the associated costs [7] and hospital stay [8].

For certain patients, harvesting autologous cells using an intraoperative setup, and enriching or depleting to obtain the appropriate cell population before delivery to the fracture location may improve regeneration. Clinical approaches using CD34+ cells in critical limb ischemia and in non-union treatment are already in place [9,10]. An intraoperative approach to cell therapy was also reported for patients with ischemic cardiomyopathy, using CD133+ cells derived from iliac crest aspirate [11], and in the context of bone regeneration using marrow-derived connective tissue progenitors [12]. In both cases, positive cell isolation used magnetic beads for cell separation, and these beads remained attached to the cells that were transplanted. In both Europe and the USA, modification of transplanted cells, which includes the use of antibodies or antibody-labeled beads that are not removed, may constitute more than a minimal manipulation and would subsequently be classified as an advanced-therapy medicinal product (ATMP), resulting in substantially greater regulatory burden for the process. It is therefore of interest to develop new isolation procedures, for both positive and negative cell isolation that would be minimally manipulative to be potentially excluded from the ATMP regulation. This would represent a platform technology for various intra-operative approaches.

The aim of this project was to develop and prototype an approach to perform cell enrichment using biophysically induced reversible cell binding. Whole blood was examined as the cell source, since no additional invasive procedure would be necessary for the patient and it could be done in parallel with a surgery. The initial target for this work is CD31, also known as Platelet/Endothelial Cell Adhesion Molecule 1 (PECAM-1), cells, which have angiogenic, osteogenic and immunosuppressive properties [13]. These cells have been used successfully in vivo for treating ischemic vascular disease [14–16]. These cells are widely available, as 70 to 78% of viable lymphocytes and monocytes express CD31 and their properties appear to be independent of gender, and preserved with aging [13]. To achieve a positive selection approach that yields a virtually "untouched" cell population (bead free, ligand free), we exploited reversible binding of cells to aptamers. Aptamers are oligonucleotides that can bind a target with a high specificity and yet, typically, a lower affinity than antibodies [17,18], and have minimal toxicity [19]. They potentially offer competitive advantages over antibodies and other protein biologics, as they are stable under long-term storage at room temperature, can be easily chemically synthesized in a reproducible manner, and can be chemically manipulated with relative ease. Aptamers have already been used in the clinic as therapeutics, and the first approved by the FDA was an RNA aptamer directed against vascular endothelial growth factor (VEGF)- 165 for the treatment of neovascular (wet) age-related macular degeneration in 2004 [20]. This approval paved the way for future approvals. In the current work, we validated a commercially available aptamer as a promising candidate. We subsequently used it for reversible binding of CD31+ cells, and report the development and optimization of a practical device and procedure for the enrichment of CD31+ cells.

## Materials and methods

### PBMC preparation

For the initial studies, peripheral blood mononuclear cells (PBMCs) were obtained from apheresis leukoreduction filters using traditional density centrifugation method. The Brigham and Women’s hospital specimen bank provided the samples from consented patients. The cell suspension was diluted with Dulbecco’s phosphate buffered saline (PBS) 1:1 before being layered onto density centrifugation medium (Ficoll-paque plus, VWR, #17-1440-02) and centrifuged for 30 min at 400 g without brake. The PBMCs layer was aspirated and rinsed twice at 200 g for 8 min to remove platelets. PBMCs were then used for subsequent aptamer validation or frozen for system optimization experiments. For magnetic bead studies and system validation, fresh whole blood samples were purchased from Research Blood Components (Boston, MA) and diluted with an equal volume of PBS + 2% fetal calf serum. PBMCs were obtained by following the manufacturer’s instruction with the Sepmate procedure (Stem Cell Technologies, #15460).

### Aptamer specificity

A commercially available biotinylated aptamer for CD31 was purchased (APTSCI, monolabeled (only biotinylated) #CD31-2196BCI, bi-labeled (biotinylated and with additional Cy5) #CD31-2196FBCI). To validate its specificity, PBMCs were resuspended at a concentration of 10^7^ cells/ml. 100 ul of cells were incubated with the biotinylated aptamer at different concentrations for 30 min at 4°C. Cells were then centrifuged for 5 min at 1200RPM and resuspended in 100 ul flow cytometry staining buffer (eBioscience, #00-4222-26). Cells were stained for CD31 (Alexa fluor 488, CD31, Biolegend, #303110) and biotin (PE anti-biotin antibody, Biolegend, #409004) before being analyzed on a BD LSRFortessa with HTS. For flow cytometric analyses of the release kinetics of the biotinylated CD31 aptamer, a version bi-labeled with additional fluorochrome Cy5 was used.

### Magnetic bead studies

To benchmark our new approach, established and commercially available bead sorting strategies were used as comparison. In certain studies, CD31+ cells were isolated by immunomagnetic cell separation (MACS System; Milteny Biotec) according to the manufacturer’s instruction using the CD31 Microbead kit (Milteny Biotec, #130-091-935). For the aptamer, magnetic cell sorting was performed using a cell isolation kit for CD31+ cells (APTSCI, #CD31-2196BCI) and following the manufacturer’s instruction.

#### In vitro characterization: Flow cytometry analysis

In order to analyze the different populations, cells were resuspended in flow cytometry staining buffer at 10^7^ cells/ml and stained for CD14 (Biolegend, #325628), CD45 (Biolegend, #304006), CD31 (Biolegend, #303116), CD3 (Biolegend, #300308) and CD19 (Biolegend, #302208) and analyzed on BD LSRFortessa. For all analysis, the initial gating on PBMCs was performed using FSC and SSC parameters. Subsequently, CD31+ levels were analyzed. To observe changes in naïve T cells, gating on CD3+ cells was performed before analyzing changes in CD31+ levels. For the CD31+ cells composition, gating was performed on CD31+ cells and composition with regards to monocytes (CD14+), T cells (CD3+) and B cells (CD19+) was evaluated.

### System assembly

We chose to develop a tube based system for cell enrichment. Microcentrifuge tubes (Pierce, #69705) served as the basis for the system. Using tweezers, polyethylene filters were removed from the tubes. Then, biopsy punches (Sklar Instruments, #96–115) were used to extract 4 mm diameter filters from 20 um cell strainers (Pluriselect, # 43-50020-03). A thin layer of cyanoacrylate glue (E-Z bond, #S-105) was applied to the base of the microcentrifuge tubes. The punched-out filter was then applied to the base of the tube and pressed slightly using tweezers. The system was then left to dry overnight at room temperature. Neutravidin agarose beads were packed within the tube: neutravidin agarose beads (Piercenet, #29204) were diluted in PBS with calcium and magnesium and filtered through a 100 um filter (Falcon, #352360). Remaining beads in the filter were then collected. The beads solution was allowed to settle before being resuspended in PBS. After a quick centrifugation step (1 min, 300 g), beads were resuspended in a solution of biotinylated aptamers and incubated for 20 min at 4°C. After incubation, the tube was centrifuged and washed twice in PBS to remove unbound aptamer. Two additional rinsing steps were performed before finally resuspending in 600 ul of binding buffer (APTSCI, #CD31-2196BCI) and transferring beads to the tube. In certain studies, unmodified and aptamer coated beads were mixed and used in the columns. Luer-lock adaptors allowed these columns to be used with BD biosciences syringes.

### Isolation procedure: From PBMCs to CD31+ enriched cell population

The PBMCs were run through the enrichment system at 50 ul/min, at a concentration of 2.5x10^7^ cells/ml in binding buffer for 10 min. During that step, the whole system was kept vertical during the duration of the experiment to alleviate issues related to cell sedimentation due to the small diameter of 1ml syringes. Another 500 ul of binding buffer was then run through at the same speed, before rinsing using 3 ml of PBS at 300 ul/min to remove unbound cells. An infusion syringe pump was used to control the flow-rate (Braintree Scientific, #BS300). The system was then disconnected from the syringe and beads were resuspended by sucking in and out of 1ml syringe with an 18GA needle (VWR, KT868280-1801). This approach dispersed the beads in solution and resulted in the release of the cells. The tube was centrifuged in a 2 ml eppendorf tubes at 300 g for 3 min to collect the released cell population. Cell viability was measured using the Muse^®^ Cell Analyzer.

### Conditioned medium studies

Conditioned medium from the initial PBMCs population and released cell population was prepared to assess their angiogenic and osteogenic potential. Cells were cultivated in EBM basal medium (Lonza, #CC3121) supplemented with 2% fetal calf serum and 1% penicillin/streptomycin (Biochrome) for the former and in DMEM low Glucose (Sigma-Aldrich) supplemented with 10% fetal calf serum and 1% penicillin/streptomycin (Biochrome) for the latter. 1.2x10^6^ cells were kept in 6 ml of medium over 24 hours. The medium was then sterile filtered and stored at -20°C until further experiments.

### In-vitro: Angiogenic potential

The angiogenic potential of the cells was assessed using a tube formation assay. 96-well plates were coated with 50 ul/well of Matrigel growth factor reduced (VWR, #354230) and transferred to a 37°C cell culture incubator to allow the gel to solidify. In the meantime, a single cell suspension of human umbilical vein endothelial cells (HUVECS, Lonza, #CC2519) between passage 3 and 5, at 2x10^5^ cells/ml was prepared. 50 ul of the cell suspension was added to each well in culture medium without angiogenesis activators or inhibitors. Cells were allowed to adhere for 20 minutes before adding 150 ul of conditioned medium. The plate was then incubated overnight. Networks of cell structures were examined under a microscope and images were taken at 10x. The total length of the network per image was evaluated using ImageJ and divided by the results obtained for the HUVECS cultured in culture medium without angiogenesis activators or inhibitors. For a positive control, the EGM bullet kit was used (Lonza, #CC3124).

### In-vitro: Osteogenic potential

Mesenchymal stromal cells were purchased from ATCC (#PCS-500-012). Cells from passage 3–4 were used for the experiments. In brief, 2.4x10^4^ cells per well were seeded in a 96 well-plate and cultivated in expansion medium for 24 hours before induction. Commercially available osteogenic differentiation medium (LifeTechnologies, #A10072-01) was purchased and diluted 1:1 with conditioned media and 100 μl medium were applied to each well with biweekly medium exchange. At the end of the study cell proliferation was evaluated using a redox-based metabolic assay (AlamarBlue Cell Viability Reagent, LifeTechnologies), and the osteogenic differentiation visualized via Alizarin Red S staining (ARS). The stained wells were rinsed repeatedly with PBS. Then, matrix-bound ARS was dissolved by addition of 10% cetylpyridinium chloride before measuring absorption at 562nm. The resulting values were normalized to the values obtained from alamar blue and divided by the values of the control cell group cultured in regular osteogenic differentiation medium.

## Results

### Validation of aptamer as a suitable CD31+ cell enrichment candidate

**Fig 2:**
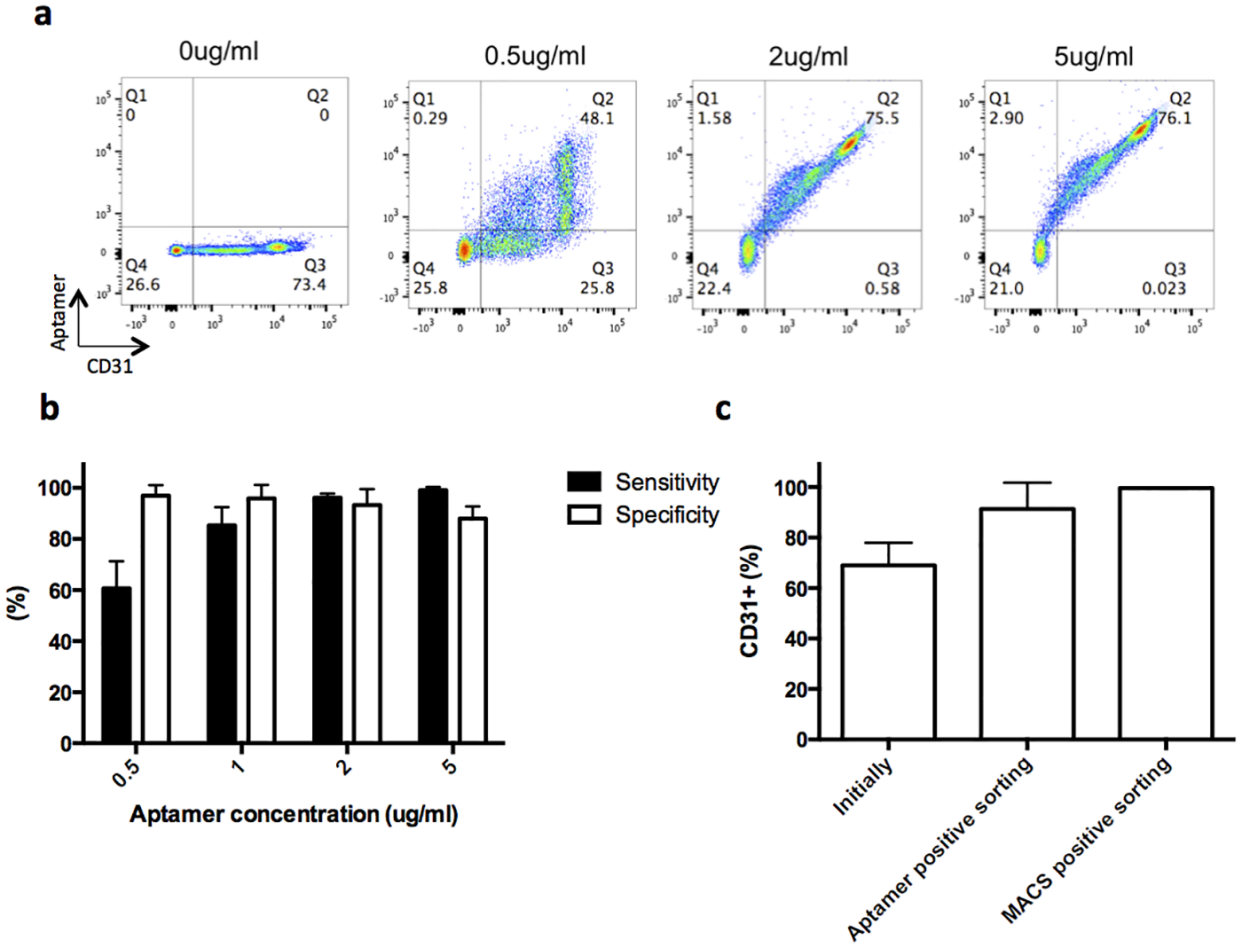
Aptamer validation with an antibody to CD31. a) Flow cytometry analysis for CD31 aptamer labeled (y-axis, aptamer) and CD31 antibody labeled (x-axis, CD31+) cells among PBMCs at different aptamer concentrations (Q1: Aptamer+CD31-, Q2: Aptamer+CD31+, Q3: Aptamer-CD31+, Q4: Aptamer-CD31-). The aptamer concentration corresponds to the individual graph title. b) Specificity and sensitivity of the aptamer for the CD31+ cells. Sensitivity was defined as the fraction of true positive (Aptamer+CD31+/CD31+). The specificity was defined as the fraction of true negatives (Aptamer-CD31-/CD31-) (n = 3). c) Fraction of cell population positively labeled with antibody to CD31 before (Initially) and after enrichment using traditional magnetic beads strategies that were coated with CD31 antibody (MACS positive sorting) and CD31 specific aptamer (Aptamer positive sorting). Beads were not released from cells prior to analysis. No significant difference was observed in post-enrichment levels of CD31 for antibody and aptamer-mediated processes (n = 4, Student’s-t-test). No error bar is visible for MACS positive sorting due to really similar high values. Values in b, c represent mean and s.d.

The ability of an aptamer to bind CD31+ cells was initially confirmed by comparing suspended cells labeled with the aptamer and a CD31 antibody. PBMCs were incubated with different concentrations of the biotinylated aptamer before staining for both CD31 and biotin using antibodies (Fig 2a). This analysis demonstrated that the aptamer was highly sensitive for the CD31 population, with the sensitivity going up to 99.0±1.3% for an aptamer concentration of 5ug/ml (Fig 2b). In addition, the specificity was also really high with values between 96.9±4.1% and 87.9±4.8 for aptamer concentration of 0.5 ug/ml and 5ug/ml respectively (Fig 2b).

To finish validating the aptamer, its capacity to concentrate CD31+ cells from a population containing a mixture of CD31 positive and negative cells was evaluated. The aptamer was coated onto magnetic beads (Ø = 1um) to allow capture of CD31+ cells (Fig 2c), and benchmarked to a commercially available magnetic isolation bead separation approach. The aptamer-based isolation and commercial antibody isolation resulted in similar enrichment of CD31+ cells.

### Development of a cell enrichment system

**Fig 3:**
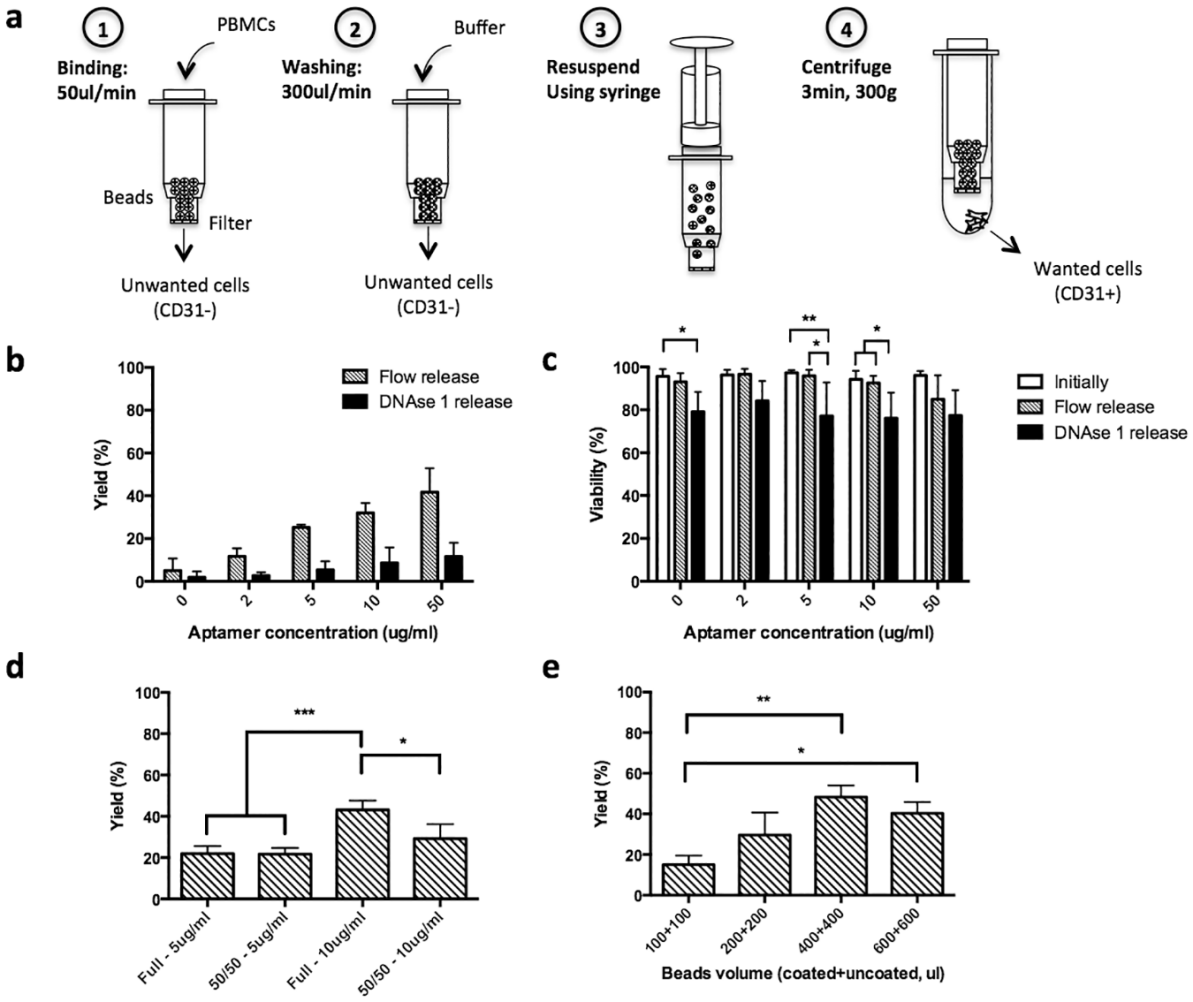
System development: Concept and optimization. a) Procedure for cell enrichment: (1) Cells are run through the system at low velocity (50ul/min) to allow binding of CD31+ cells and removal of CD31- cells that pass through the system unimpeded (2) Any remaining non-adherent cells are removed using PBS buffer wash (300ul/min) (3) Cells are dissociated from beads by resuspension using a syringe. This results in dissociation of the adherent cells from the beads (4) Tube is centrifuged immediately to collect desired cells (CD31+) that were released from beads. b) Yield as function of aptamer concentration and release type (Flow or DNAse 1 release). Yield was defined as the number of cells collected after enrichment divided by the number of cells that went through the tube. All the beads were aptamer coated and 800ul of initial neutravidin agarose bead solution was used per column. DNAse 1 was used at 500ug/ml for subsequent release (n = 3). c) Effect of release type and aptamer concentration on cell viability (n = 3, data were analyzed using two-way analysis of variance (ANOVA), * = P<0.05, **P<0.01) d) Effect of mixing uncoated beads and aptamer coated beads on yield, for two aptamer concentrations (5 and 10ug/ml). Full indicates that all beads were aptamer coated whereas 50/50 indicates that only half of the beads were aptamer coated (n = 3, data were analyzed using one-way analysis of variance (ANOVA), * = P<0.05). e) Yield as a function of initial bead volume suspension. An aptamer concentration of 10ug/ml was used for aptamer-coated beads. Data are given as A+B where A is the volume of uncoated beads, and B is the volume of coated beads (n = 3, data were analyzed using one-way analysis of variance (ANOVA), **P<0.01). Values in b, c, d and e represent mean and s.d.

The general principle of the system was as follows: PBMCs that contain a mixture of CD31- and CD31+ cells were run through the system. The CD31+ cells adhered to the aptamer coated beads while CD31- flowed through and were discarded. CD31+ cells were then released from beads by imposing fluid shear with a simple mixing of the beads with attached cells and additional medium. Cells released were then collected by centrifugation (Fig 3a).

First we optimized the aptamer concentration for the coating of the beads. Increasing the aptamer concentration resulted in an overall increase in the capture of cells (yield), as measured using a biophysically induced (flow release) and a subsequent DNAse based release (Fig 3b). In general no significant difference was observed between the initial cell viability and that of flow-released cells (Fig 3c). However, at the highest aptamer concentration, flow-based separation did result in a decrease in cell viability after release, likely due to the overall stronger avidity between the cells and these beads. To determine if adherent cells were retained after intended release, we applied DNAse 1 to digest aptamers after flow release. The use of DNAse 1 significantly affected cell viability at all conditions and increasing aptamer concentration led to an increase of retained cells (S1 Fig). Importantly, the flow based release strategy resulted in an enrichment of CD31+ cells at a lower aptamer concentration but not at the highest aptamer concentrations (S1 Fig). Therefore, subsequent steps for optimization were focusing on aptamer concentrations of 5 ug/ml and 10 ug/ml, as these provided an appropriate compromise between cell yield, CD31+ purity and cell viability.

The next set of studies explored the impact of combining uncoated and aptamer coated beads, and varying the total bead number in the columns. We employed a mixture of uncoated beads and aptamer coated beads in the columns in the first studies, as the system sometimes clogged in the earlier studies and we hypothesized this was due to cells binding throughout the device. An equivalent yield to columns containing all coated beads was found with a 50:50 mix of coated:uncoated beads at the aptamer concentration of 5 ug/ml, but the yield was significantly reduced at an aptamer concentration of 10ug/ml for the combination of uncoated and coated beads (Fig 3d). However, there was no statistical difference using a 50:50 mix between 5 and 10ug/ml. Therefore, we use a 5ug/ml concentration for system validation. With the 50:50 mix, clogging was no longer observed, avoiding issues related to pressure building-up in the system. The incorporation of uncoated beads also facilitated resuspension of the beads during the cell recovery, although this effect was not quantified.

The final parameter optimized was the bead volume. An increasing cell yield was initially obtained as the bead number was increased, until a saturation behavior was observed at 400 ul of bead volume and above (Fig 3e). Importantly, microscopy-based observation of the cell population enriched with this system revealed none of the isolated cells contained bound beads.

### System validation

**Fig 4:**
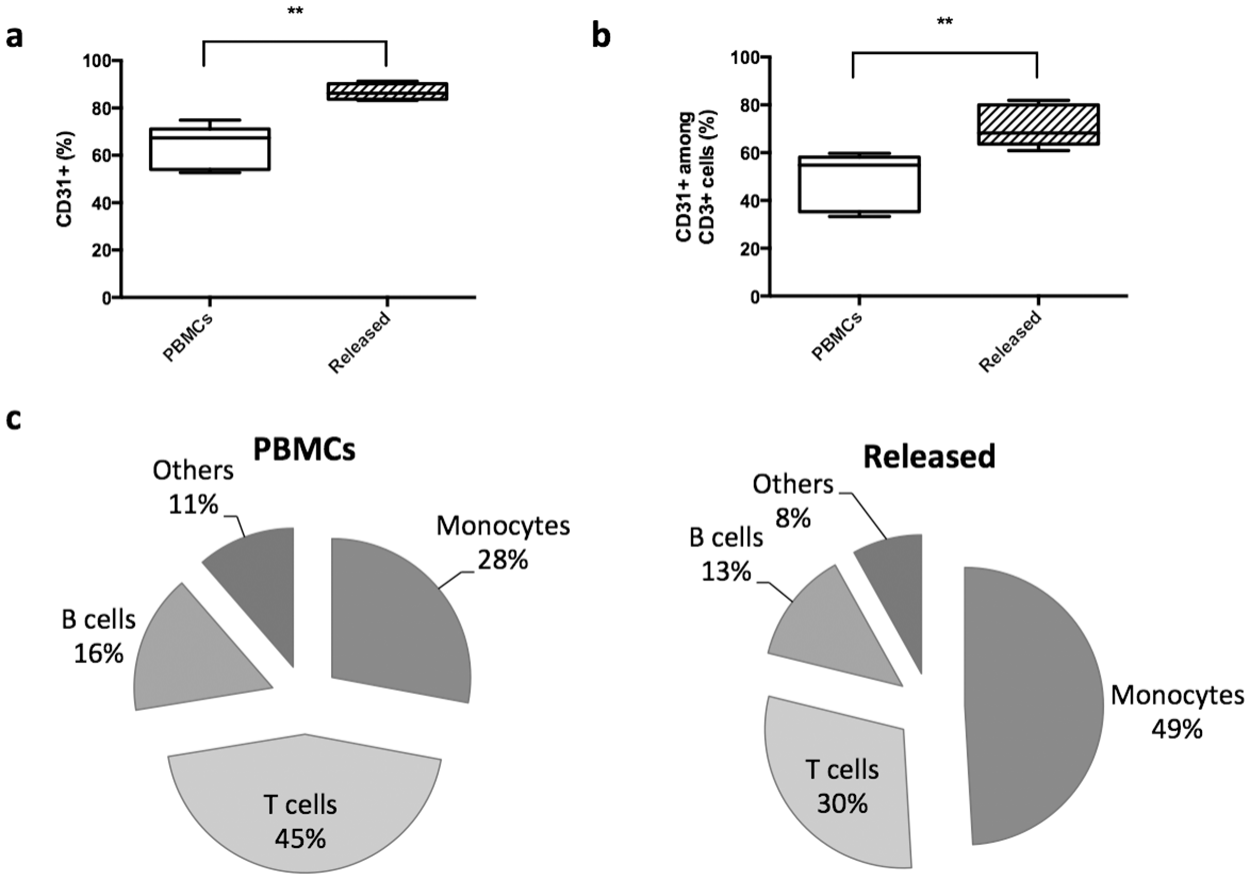
System validation using fresh whole blood samples. The experiments were performed using a 5ug/ml aptamer concentration with an initial volume of 800ul of neutravidin agarose beads. Half the beads in columns were aptamer coated. a) The percentage of PBMCs that are CD31+, as indicated by antibody staining and FACs analysis, before (PBMCs) and after enrichment using the aptamer-bead column system (Released) b) Percentages of cells in the overall population that were CD3+CD31+ (naïve T cells), as indicated by antibody staining and FACS analysis, before (PBMCs) and after enrichment (Released) c) Composition of the CD31+ fraction according to antibody staining and FACS analysis before (PBMCs) and after enrichment (Released) (n = 5, values in a and b represent mean and s.d., values in c represent mean, data were analyzed using paired Student’s t-test, ** = P<0.01, ***P<0.001).

The next studies quantified the ability of the optimized system to enrich CD31 cells from fresh PBMCs derived from probands whole blood samples. The column used in these studies utilized beads coated with 5 ug/ml of aptamer, a 50/50 ratio of coated to uncoated beads, and a total bead volume of 800 ul (400 μl uncoated and 400 μl aptamer coated). The use of these columns resulted in a significant increase in the percentage of isolated cells that were CD31+, from 64±9 to 87±3%, while retaining a high cell viability of 97±1% (Fig 4a). The yield of the CD31+ cells was 25.1±0.9%. Further analysis revealed that the CD31+ fraction was significantly enriched in monocytes (CD31+CD14+), as compared to the initial PBMC population (Fig 4c). A significant increase in naïve T cells (CD3+CD31+) was also observed (Fig 4b). For the precise gating strategy please refer to the material and methods section.

**Fig 5:**
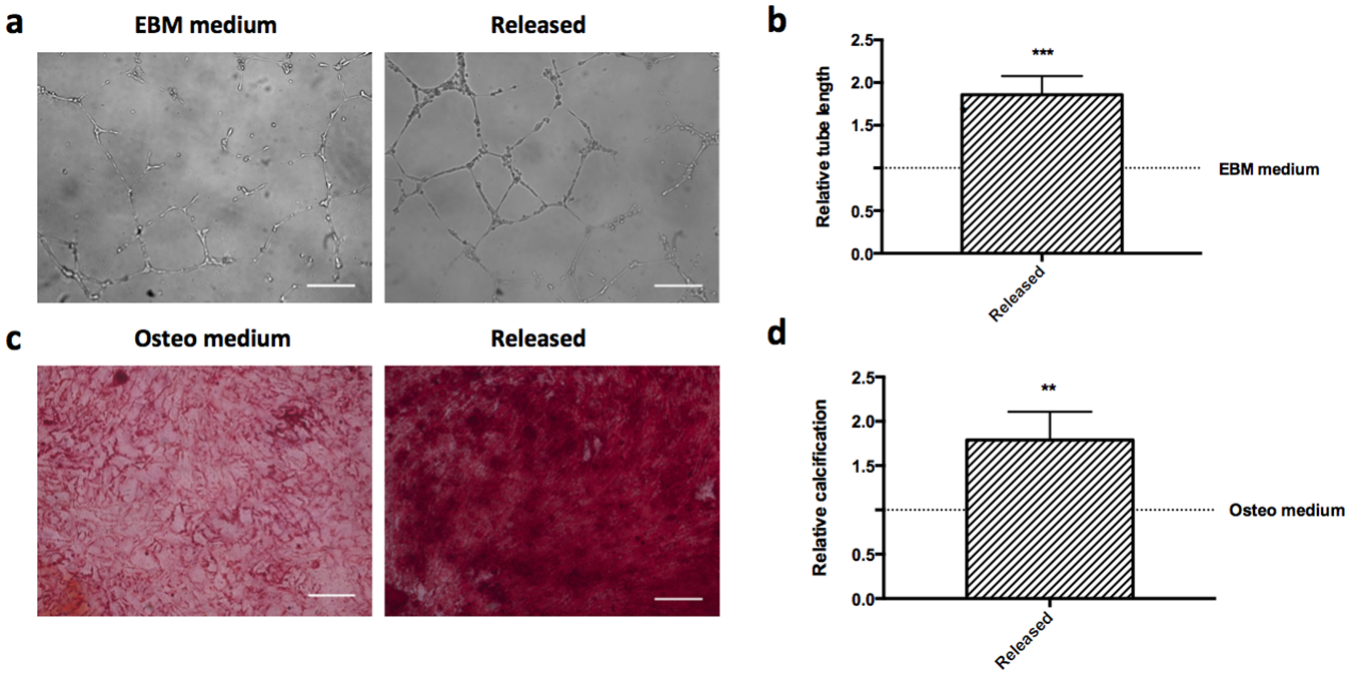
Angiogenic and osteogenic properties of released cell population. Conditioned medium was prepared from cell populations enriched using a 5ug/ml aptamer concentration with an initial volume of 800ul of neutravidin agarose beads. Half the beads were aptamer coated. a) HUVECs cultured in endothelial basal medium without addition of angiogenesis activators (EBM medium) or with conditioned medium (50ul EBM medium + 150ul conditioned medium) from released cell population (Released) were observed using bright field microscopy. b) Relative tube length was calculated and defined as the mean total length of the network for released samples divided by the results obtained for the HUVECS cultured in EBM medium. c) Alizarin red staining of MSCs differentiated for two weeks in osteogenic medium (Osteo medium) or in conditioned medium (½ osteogenic medium + ½ conditioned medium, Released). d) Relative calcification was calculated and defined as ratio between absorption values obtained by dissolution of matrix-bound ARS using 10% cetylpyridinium divided by values obtained from alamar blue, and normalized to the values obtained for the osteo medium group. (n = 5, scale bar = 200um). Values in b and d represent mean and s.d., data were analyzed using paired Student’s t-test, **P<0.01, ***P<0.005).

To confirm the previously reported regenerative potential of enriched CD31+ cells [13] when obtained using the optimal column conditions, both an osteogenic and angiogenic in vitro assay were utilized. An in vitro tube formation assay validated the increased angiogenic potential of the enriched cell population; the total length of capillary-like tubes was increased 1.9±0.2 fold, as compared to the control condition (Fig 5a/5b, S3 Fig). The osteogenic potential was also confirmed, as conditioned medium from enriched cells increased calcification by a factor of 1.8±0.3 in MSCs, as compared to the control condition (Fig 5c/5d, S3 Fig). Further data of control groups and analyses of the subpopulations have previously proven the high regenerative potential of the total CD31+ population in vitro and in vivo [13], based on which a simplified in vitro assessment was performed here.

## Discussion

The goal of this study was to evaluate the potential of a cell sorting strategy that would allow to sort for beneficial or detrimental cells from whole blood. Specifically, we aimed at developing an approach for positive cell isolation that would result in a clean (bead free) cell population enriched for a specified cell surface marker. We prototyped a system to perform biophysically induced reversible binding and validated it using CD31+ cells as an example.

The commercial aptamer showed high sensitivity and specificity for CD31+ cells derived from PBMCs. Flow cytometry data showed mainly double positive (CD31+ and Apt+) and double negative populations (CD31- and Apt-) for cells isolated using aptamer concentrations of 2ug/ml and above. Also, magnetic isolation bead separation resulted in similar enrichment for aptamer and antibody based isolation. This is in agreement with recently published data using the same aptamer to target another cell type expressing CD31; endothelial progenitor cells were successfully isolated from a mixture with CD31- 293FT cells at a comparable level to antibody isolation [21]. In this work, we validated the aptamer for the first time for PBMCs.

Cell release from this enrichment system was mediated solely by mechanical forces and did not require the use of any additional chemicals that could contaminate the isolated cells. A compromise between cell yield, CD31+ purity, and cell viability was found by adjusting aptamer concentration, bead number and combining uncoated and aptamer coated beads. In current commercial approaches, cell release from beads in antibody approaches utilizes an elution buffer, and antibodies remain attached to the cells. Whereas aptamers offer the possibility of a reversible binding approach, strategies reported to date typically require exposure to the aptamer complementary strand, enzymatic treatment [22], or use of a high concentration dextran sulfate [20]. Chemical-free approaches to aptamer binding have been reported in which the temperature and shear sensitivity of aptamer binding has been exploited in microfluidic devices [23,24]. However, to the authors’ knowledge, this is the first report of reversible aptamer binding without the use of any chemical or temperature modification. As biological bonds based on secondary interactions can be sensitive to applied stress [25–28], it is not surprising that shear stresses induced by resuspending the beads can be sufficient to induce cell release. Additionally, by resuspending the beads and separating them, there is a loss of the avidity effect coming from multiple beads in contact with the cells and the affinity of individual aptamer might be too weak to hold the cells for long. As some cells still remained attached to the beads after shearing, as evidenced by their recovery with a subsequent DNAse step, higher shear stresses could potentially release a greater number of CD31+ cells from the agarose beads. However, increased shear could also lead to cell damage and loss of cell viability. The aptamer affinity was reported to be significantly lower (Kd = 1.14x10^-9M) than the extremely high affinity biotin-neutravidin linkage (Kd = 10^-14 M). Therefore, it is also reasonable to assume that bead-free cells were also aptamer-free. This was confirmed using a bi-labeled version of the biotinylated aptamer with additional fluorochrome Cy5 (S4 Fig). This approach gave the possibility to analyze the cells once collected after enrichment, without having to do any additional labeling step that might result in aptamers falling off or unspecific binding of the secondary antibody. Also, according to the manufacturer, there was no biophysical differences between the mono- and the bi-labeled aptamer.

Importantly, the released cell population with the system developed in this report is free of beads, likely due to the size difference between the beads and the mesh size of the filter component. Neutravidin agarose beads, with a diameter initially between 45 and 165 um, were filtered through a 100 um cell strainer to select only larger size beads. Concerns from regulatory offices often relate to the possibility of remaining beads in the final product, since most of the cell sorting options currently available rely on beads in the nanometer to micrometer range. However, the filters at the base of the tubes in the current system have a 20 um mesh size that prevent bead passage, but was still adequate for leukocytes, since their diameter is between 6 and 10um [29,30]. The possibility of phagocytosis of immunobeads during positive cell isolation is also a major concern [31], but is not an issue in our approach due to the fact that the beads used here are several times larger than the cells creating a clear size separation between cell and capturing beads as was also reported for other systems before [32].

The system leads to an enriched CD31+ cell population with increased angiogenic and osteogenic potential. A particular enrichment in monocytes was noted, which is not surprising since we observed that they had the highest level of CD31 expression among PBMCs. However, an increase in naive T cells also took place. In a previous study, a similar concept of selective enrichment was reported by Sass et al. but using the magnetic activated cell sorting [13]. The conditioned medium from the cell population enriched with this improved system led to an increase in the total tube network length in an angiogenesis assay, and enhanced the osteodifferentiation of MSCs. Those results are in agreement and have been already demonstrated by Sass et al. for CD31+ enriched cell populations and the respective subpopulations [13]. Other studies have also shown that monocytes are involved in promoting bone formation through non-immunological aspects either directly by cytokine secretion of osteogenic and angiogenic factors, or indirectly by activating additional cells to also secret appropriate cytokines [33,34]. However, angiogenic and osteogenic promotion are not exclusive to monocytes among CD31+ cells, and the enrichment of naïve T cells may also underlie these effects. Indeed, upon stimulation, CD31+ T cells had an increased secretion of angiogenic factors (G-CSF, interleukin 8 and matrix metallopeptidase-9) compared to CD31- T and those cytokines are highly involved in vessel development and damage response to tissue ischemia. [35].

This system has significant therapeutic potential. It is commonly accepted that 10^6^ PBMCs/ml can be isolated from blood [36]. This would imply that with the current approach, approximately 15.8 million enriched CD31+ cells could be collected from 100 ml of blood, which is a reasonable blood draw. Further development of this system could also allow it to be utilized to target other surface antigens for either enrichment or depletion. Methods to generate an aptamer to a target of interest have been previously reported [37], and allow for the in vitro evolution of nucleic acid molecules with highly specific binding to target molecules. To utilize this system to deplete undesirable cell populations, one could simply use agarose beads coated with an appropriate antibody or aptamer to remove these cells from a mixed population; in this approach, the desirable cells would be those that initially pass through the system. One target for selective depletion could be CD8+ T cells, as they have been shown to have a negative impact on the bone healing process [38]. Coating the agarose beads with a biotinylated antibody for CD8+ allowed depletion of those cells from a starting PBMCs population (S2 Fig). One could also combine a series of these separations, allowing both positive and negative selections to be integrated into one system.

## Supporting information

S1 Fig a) Percentages of cells in the overall PBMCs population that were CD31+, as indicated by antibody staining and FACs analysis, as a function of aptamer concentration and release type (Flow or DNAse1 release). CD31+ levels were compared before (Initially) and after procedure at two aptamer concentrations (10 and 50ug/ml) for the two releases (Flow or DNAse 1). b) Impact of procedure on cell viability. Cell viability as determined by Muse^®^ Cell Analyzer, was evaluated in the initial PBMCs population (Initially) and in released cell population at two aptamer concentrations (10 and 50ug/ml) for the two releases (Flow or DNAse 1), n = 3. Data were analyzed using one-way analysis of variance (ANOVA). All beads were aptamer coated for this experiment.
(TIF)

S2 Fig Adaptation of the system towards cell depletion by coating beads with a biotinylated CD8 antibody instead of a CD31 aptamer. Beads were incubated for 20 min at 4°C with a biotin anti-human CD8 antibody (Biolegend, #344720). PBMCs were run through the system and non-adherent cells collected and analyzed. Histograms from FACS analysis for CD8+ cells, determined using antibody to CD8, in both the original cell population (PBMCs) and collected cells (Depleted population).
(TIF)

S3 Fig Biological properties of released cell population. Conditioned medium was prepared from PBMCs and enriched CD31+ cells using a 5ug/ml aptamer concentration with an initial volume of 800ul of neutravidin agarose beads. Half the beads were aptamer coated. a) Relative tube length was calculated and defined as the mean total length of the network formed by HUVECS cultured under conditioned medium derived from PBMCs and Released (CD31+) cells (n = 5), normalized to the values obtained for the HUVECS cultured in EBM medium without growth factor addition (indicated as dotted line). EBM medium plus additional growth factors (EBM bullet Kit, Lonza) served as a positive control. CD31+ released cells had a significant higher impact on angiogenic tube formation than the whole PBMC fraction b) Impact on osteogenic differentiation and matrix calcification was calculated and defined as the ratio between absorption values obtained by dissolution of matrix-bound ARS using 10% cetylpyridinium divided by values obtained from alamar blue, and normalized to the values obtained for the osteo medium group (n = 3). DMEM Expansion medium containing 10% FCS served as a negative control, DMEM diluted with osteo medium, eventually containing 5% FCS served as FCS adapted control. Values in a and b represent mean and s.d., data was analyzed using Anova-One way with Bonferroni’s comparison of selected groups, * significant to control, # significant to Released CD31+, *P<0.05, **P<0.01, ***^/###^P<0.005).
(TIF)

S4 Fig Release of the aptamer. Flow cytometric analyses after cell enrichment using a Cy5-coupled version of the biotinylated aptamer were performed. Cells were analyzed before processing as negative control, the released cells were analyzed prior to a re-newed staining to show that none of the Cy5-fluorochrome-coupled aptamer remained on the cells and then re-stained and analyzed again to evaluate the median fluorescence intensity of aptamer coupled cells. The Histogram in a) shows representative data from 1 patient. The orange line represents the unprocessed, unstained sample as a negative reference (median fluorescence intensity 21 AU). The red line represents the fluorescence intensity of the released cell population (median fluorescence intensity 52,4 AU), the blue line shows the median fluorescence intensity after renewed staining with the Cy5-fluorochrome-couple aptamer after processing (median fluorescence intensity 1044 AU), b) shows the average median fluorescence intensity (MFI) from before and after the enrichment of cells (negative reference MFI 42,6 ± 18,77 AU, released cell population MFI 31,13 ± 18,42 AU, released and re-stained MFI 939 ± 167,36 AU) (n = 3, ***P<0.0001, Anova-One way with Bonferroni’s comparison).
(TIF)
