# Supplementary figures and images for: Biophysical induction of cell release for minimally manipulative cell enrichment strategies
Source: PLoS One. 2017 Jun 30;12(6):e0180568. doi: 10.1371/journal.pone.0180568 (PMC5493423; doi:10.1371/journal.pone.0180568)

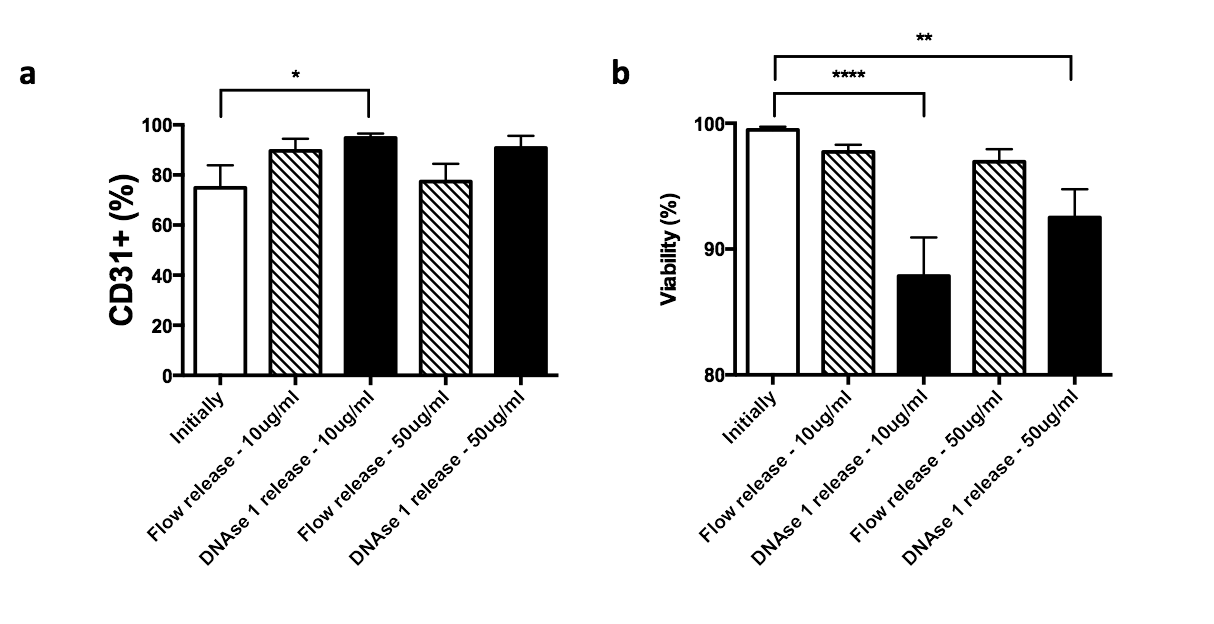

Supplement: S1 Fig — a) Percentages of cells in the overall PBMCs population that were CD31+, as indicated by antibody staining and FACs analysis, as a function of aptamer concentration and release type (Flow or DNAse1 release). CD31+ levels were compared before (Initially) and after procedure at two aptamer concentrations (10 and 50ug/ml) for the two releases (Flow or DNAse 1). b) Impact of procedure on cell viability. Cell viability as determined by Muse® Cell Analyzer, was evaluated in the initial PBMCs population (Initially) and in released cell population at two aptamer concentrations (10 and 50ug/ml) for the two releases (Flow or DNAse 1), n = 3. Data were analyzed using one-way analysis of variance (ANOVA). All beads were aptamer coated for this experiment. (TIF) [file pone.0180568.s001.tif]

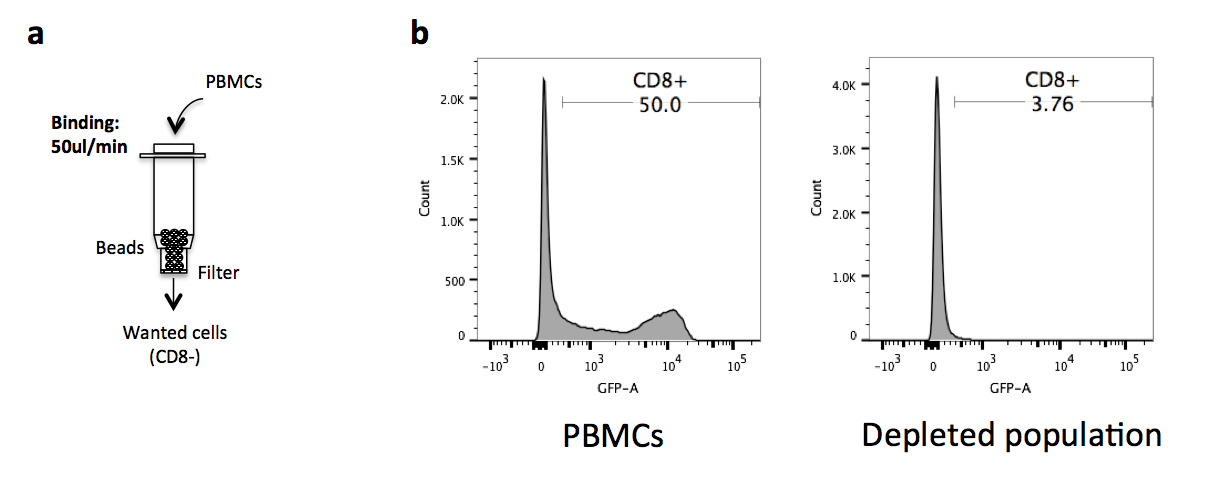

Supplement: S2 Fig — Beads were incubated for 20 min at 4°C with a biotin anti-human CD8 antibody (Biolegend, #344720). PBMCs were run through the system and non-adherent cells collected and analyzed. Histograms from FACS analysis for CD8+ cells, determined using antibody to CD8, in both the original cell population (PBMCs) and collected cells (Depleted population). (TIF) [file pone.0180568.s002.tif]

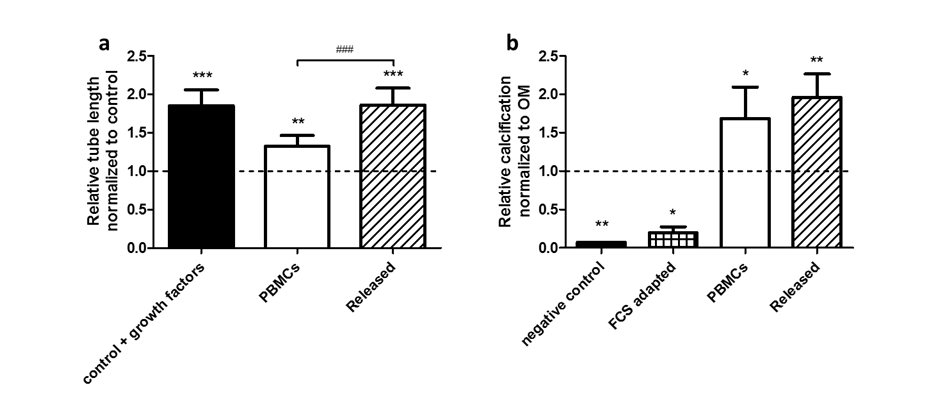

Supplement: S3 Fig — Conditioned medium was prepared from PBMCs and enriched CD31+ cells using a 5ug/ml aptamer concentration with an initial volume of 800ul of neutravidin agarose beads. Half the beads were aptamer coated. a) Relative tube length was calculated and defined as the mean total length of the network formed by HUVECS cultured under conditioned medium derived from PBMCs and Released (CD31+) cells (n = 5), normalized to the values obtained for the HUVECS cultured in EBM medium without growth factor addition (indicated as dotted line). EBM medium plus additional growth factors (EBM bullet Kit, Lonza) served as a positive control. CD31+ released cells had a significant higher impact on angiogenic tube formation than the whole PBMC fraction b) Impact on osteogenic differentiation and matrix calcification was calculated and defined as the ratio between absorption values obtained by dissolution of matrix-bound ARS using 10% cetylpyridinium divided by values obtained from alamar blue, and normalized to the values obtained for the osteo medium group (n = 3). DMEM Expansion medium containing 10% FCS served as a negative control, DMEM diluted with osteo medium, eventually containing 5% FCS served as FCS adapted control. Values in a and b represent mean and s.d., data was analyzed using Anova-One way with Bonferroni’s comparison of selected groups, * significant to control, # significant to Released CD31+, *P<0.05, **P<0.01, ***/###P<0.005). (TIF) [file pone.0180568.s003.tif]

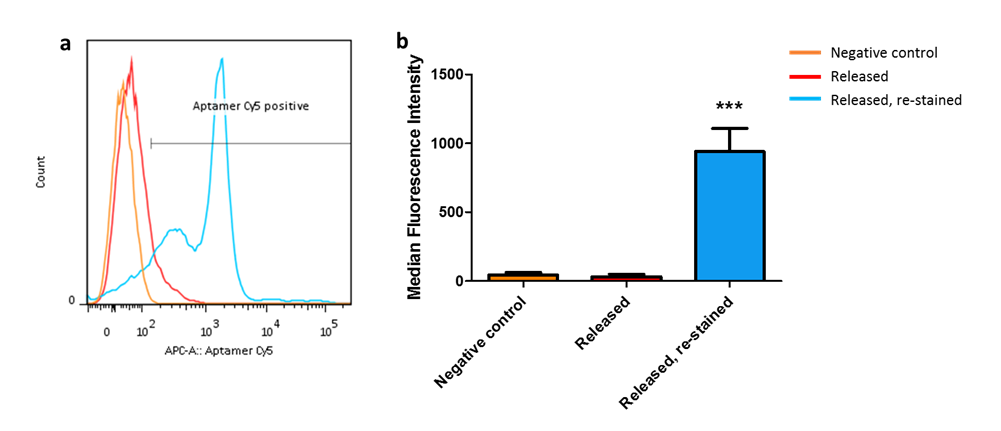

Supplement: S4 Fig — Flow cytometric analyses after cell enrichment using a Cy5-coupled version of the biotinylated aptamer were performed. Cells were analyzed before processing as negative control, the released cells were analyzed prior to a re-newed staining to show that none of the Cy5-fluorochrome-coupled aptamer remained on the cells and then re-stained and analyzed again to evaluate the median fluorescence intensity of aptamer coupled cells. The Histogram in a) shows representative data from 1 patient. The orange line represents the unprocessed, unstained sample as a negative reference (median fluorescence intensity 21 AU). The red line represents the fluorescence intensity of the released cell population (median fluorescence intensity 52,4 AU), the blue line shows the median fluorescence intensity after renewed staining with the Cy5-fluorochrome-couple aptamer after processing (median fluorescence intensity 1044 AU), b) shows the average median fluorescence intensity (MFI) from before and after the enrichment of cells (negative reference MFI 42,6 ± 18,77 AU, released cell population MFI 31,13 ± 18,42 AU, released and re-stained MFI 939 ± 167,36 AU) (n = 3, ***P<0.0001, Anova-One way with Bonferroni’s comparison). (TIF) [file pone.0180568.s004.TIF]
